# The Association Between Family Flexibility, Food Preoccupation and Body Image Among Crystal Abuser Women

**DOI:** 10.5812/ijhrba.7503

**Published:** 2012-11-26

**Authors:** Masoumeh Rahmatizadeh, Anahita Khodabakhshi Koolaee

**Affiliations:** 1 Department of Clinical Psychology, Elmo Farhang University, Tehran, IR Iran; 2 Departments of Clinical Psychology, Social Welfare & Rehabilitation University, Tehran, IR Iran

**Keywords:** Methamphetamine, Drug Abuse, Body Image, Women

## Abstract

**Background:**

Methamphetamine (MA) is a highly addictive stimulant which has destructive effects. There is also evidence that methamphetamine use in some females, partly is due to their desire to lose weight. The purpose of this study was to determine the association between family flexibility, food preoccupation and body image among crystal abuser women.

**Objectives:**

This study tried to evaluate whether food preoccupation, body image and family flexibility affect on crystal abuse in women.

**Patients and Methods:**

Eighty crystal abuser women were chosen with convenience sampling and they responded to instrument of body image (Fisher), family flexibility (Olson) and food preoccupation (Tapper) questionnaires.

**Results:**

There is a significant association between family flexibility and food preoccupation (P < 0.01) and also other variables; there is no significant association between family flexibility and body image, body image and food preoccupation (P > 0.05). Also, family flexibility is prediction of food preoccupation (P < 0.01).

**Conclusions:**

These results emphasize that the components of eating behaviors (e.g. food preoccupation) and family flexibility can be considered in therapeutic interventions (prevention and treatment) for women to crystal withdrawal.

## 1. Background

Methamphetamine, a stimulant colloquially known as ‘crystal meth’, ‘crank’, ‘ice’, ‘chalk’ or ‘Tina’, is a highly addictive substance which can be snorted, smoked, ingested orally or rectally and injected. Amphetamine is powerful a psycho-stimulant which directly affect the autonomic and central nervous systems even in small amounts (1). Data from neuroimaging studies, neuropsychological testing, and psychiatric evaluation indicates that heavy use of MA contributes to a variety of psychiatric pathologies, including psychosis, mood disturbance, suicidal ideations, anxiety, hostility, psychomotor dysfunction, deficits in cognitive skills, and, in extreme cases, paranoia, hallucinations, and delusion (2). Immediately after ingestion of MA, users experience a number of highly desirable sensations, including a sense of euphoria caused by an elevated level of dopamine. Other desirable sensations associated with MA include increased productivity, heightened attentiveness and curiosity, hyper sexuality, decreased anxiety, and increased energy. The euphoric feelings vary in intensity and duration, depending on the route of administration, with smoking or intravenous injecting leading to intense, but brief, euphoria and with oral ingestion or snorting leading to a slightly less intense, but more long-lasting (2). Previous findings showed that Substance use disorders often co-occur with eating disorders in female populations (3). Substances associated with eating disorders include alcohol, tobacco, cannabis, cocaine, heroin and amphetamines (4). Higher use among girls is consistent with other researches reporting gender differences in methamphetamine use. In a review of publications on methamphetamine over the past 30 years, Dluzen and Liu found that women tend to begin methamphetamine use at earlier ages, appear more dependent on it, but also respond better to treatment than do men. Methamphetamine use appears to be associated with depression in women, and women seem more committed to the drug, whereas men are more likely to use other drugs in the absence of access to methamphetamine. There is also evidence that methamphetamine use in some females, partly is due to their desire to lose weight (5). In a study focusing on gender differences in drug use history among a broad cross-section (n = 350) of former clients from a large publicly funded treatment system, Brecht et al. found that initial methamphetamine tendency in females to lose weight is five times more than males (36% vs. 7%) (6). Patients with bulimia nervosa are also more likely to report illicit drug use, particularly amphetamines, barbiturates, marijuana, tranquilizers and cocaine (4). Because of an emphasis on diet and impotence in different social levels, factors associated with weight loss such as a body image require more attention (7) Body image is a ‘‘multifaceted psychological experience of embodiment’’ which encompasses evaluative thoughts, beliefs, feelings, and behaviors related to one’s own physical appearance (6) and body image can be influenced by factors such as physical growth, individual interactions with social environment, accidents, injuries (8). On the other hand, the prevalence of body dissatisfaction is a major concern, because defects and mental disorders such as low self-esteem, depression, social anxiety, eating disorders, sexual disorders and diseases are associated with body deformity (9). The most popular approach among people for negative body image is weight loss (10). Overestimation of weight among normal weight adolescents and accurate perceptions of weight among overweight adolescents were associated with higher rates of eating behaviors disorders. In normal weight adolescents, use of all three substances (tobacco, binge drinking, and cocaine) was associated with each disordered eating behavior (11). On the other hand, according to the results of psychological researches, diet can lead to food preoccupation and food deprivation, therefore, food Preoccupation is characteristic of those diets to lose weight. It is important for several reasons. First, it is believed to impair performance on a range of cognitive tasks. Compared to nondieters, dieters have been shown to display impaired performance on tests of reaction time, sustained attention and immediate free recall (12). According to Johnson and Bedford, foods preoccupation starts early in life and does not change as one ages. Many weight concerned middle age women could have developed food preoccupation in their youth while young weight-concerned women have not fully developed an eating problem (13). Cognitive food preoccupation can also lead to excessive eating or emotional eating. This type of eating is not as severe as binging but can also lead to obesity. A research performed on the eating disorder population has found that cognitive factors such as cognitive distortions, cognitive food preoccupation, and cognitive avoidance of emotion are all underlying factors which are strongly associated with development and maintenance of an eating disorder (14). Also, the important role of family as the primary educator of social values, in the development of eating disorders should not be ignored (7). In family systems theory, the effects of dynamics within the family have been shown to be as a significant risk factor for developing anorexia nervosa. Among these components, the component of flexibility has an important role; “family flexibility is the amount of change in its leadership, role relationships, and relationship rules” (15). The results suggest that the transactional characteristics of an anorexic patient’s family organization include enmeshment, rigidity, overprotectiveness, and lack of conflict resolution and interactions avoiding conflicts (16, 17). In fact, several researches and studies have been performed on the factors which affect addiction as social, economical as well as cultural factors, despite the fact there is a gap in the study of precursor psychological and personality factors affecting addiction, so it was tried to pave a new way to this subject in this study.

## 2. Objectives

This study tried to evaluate the association between food preoccupation, body image and family flexibility in crystal abuser women.

## 3. Patients and Methods

### 3.1. Participants and Plan

This study was conducted in 2011 in Tehran. Eighty crystal abuser women volunteers from addiction treatment centers were chosen. Age ranged from 15 to 40 years. Inclusion criteria were as follow:

1) Being a crystal abuser or used crystal as main substance with another opiate or nonopiate.

2) Without any severe mental and physical illnesses.

3) High school level as the minimum level of education.

4) Time of use for at least 6 months.

The participants responded to the questionnaires knowing that their information would be remained confidential.

### 3. 2. Measurements

### 3. 2. 1. Food Preoccupation (12)

Questionnaire began with a series of 26 statements relating to thoughts about food. Of these, 3 were designed to assess thought frequency, and 23 for emotional valence of thoughts, answers could be either positive (9 items), negative (9 items) or neutral (5 items). Participants were asked to rate the extent to which they agreed or disagreed with each statement on a 5 point scale (‘completely disagree’, ‘disagree a bit’, ‘neither agree nor disagree’, ‘agree a bit’, ‘completely agree’. Four items were reverse scored. The alpha coefficients for thought frequency, positive statement, negative statement and neutral statement were reported as 0.87, 0.81, 0.80, 0.73. Validity of this instrument in community sample of 30 people of crystal abuser women was obtained by the author as follow: for thought frequency, positive statement, negative statement and neutral statement is 0.79, 0.76, 0.71, and 0.73. The Food Preoccupation Questionnaire could be a useful tool for exploring links between food-related cognitions and behavior (12).

### 3.2.2. Body Image

Body image was designed by Fisher in 1970. This questionnaire is a 46-item self-report questionnaire. Responses were rated on a 5-point Likert-type scale, ranging from 1 (completely disagree) to 5 (completely agree). The Cronbach alpha reliabilities is 0.84.

### 3.2.3. Family Flexibility

This questionnaire was inspired by Circumplex Model of Olson (15) which is made by Shakeri. Questionnaire is a series of 16 statements, Responses were rated on a 5-point Likert - type scale, ranging from 1 (completely disagree) to 5 (completely agree). The Cronbach alpha reliabilities is 0.89. In many researches Olson’s family flexibility questionnaire is used to determine family flexibility value (18).

### 3.3. Procedure

All participants completed 3 questionnaires, including Body Image, Family Flexibility and Food Preoccupation questionnaire. Then data collected was analyzed with SPSS-16 software. Inferential statistics, Pearson moment correlation, correlation matrix and regression were used to analysis data.

## 4. Results

Is there a significant association between family flexibility and body image in crystal abuser women? For responding to this research question, a Pearson correlation coefficient was conducted on the data, the results are displayed in *Table 1* and *Table 2*, which shows that correlation coefficient between body image and flexibility is not significant (r = - 0.07, P < 0.01). The second research question was “Is there a significant relationship between body image and food preoccupation?”

**Table 1:** General Characteristics of Crystal Abuser Women in Percent

	F	CF
**Age Group, y**		
15 - 20	10	10
21 - 25	25	35
26 - 30	26.2	61.2
31 - 35	18.8	80
36 - 40	20	100
**Education**		
High school	46.2	46.2
Diploma	38.8	85
Baccalaureate degree	7.5	92.5
Master’s	7.5	100
**Marital status**		
Single	30	30
Married	35	65
Divorced	30	95
Widowed	5	100

Abbreviations: F, frequency; CF, cumulative frequency

**Table 2:** Correlation Matrix Between Family Flexibility with Food Preoccupation and Body Image

	Body Image	Family Flexibility	Food Preoccupation
**Body image**	-		
**Family flexibility**	-0.07	-	
**Food preoccupation**	0.09	-0.32 ^a^	-

^a^P ≤ 0.01

Our findings suggest that correlation between body image and food preoccupation was not statistically significant (r = 0.09, P < 0.01). The third research question was” Is there a significant association between family flexibility and food preoccupation?” The correlation coefficient between two variables flexibility and food preoccupation represents a significant negative relationship between them (r = - 0.32, P < 0.01). The fourth research question was “Does the family flexibility predicts food preoccupation and body image in this group?” The regression test for each predictor was separately used to predict food preoccupation and body image from family flexibility which its results are displayed in *Table3*.

**Table 3:** Summary of the Regression Model of Family Flexibility on Food Preoccupation in Crystal Abuser Women

	SS	df	MS	F	P value	R	R2
**Regression**	2.54	1	2.54	9.15	0.006	0.32	0.093
**Residual**	21.70	78	0.28				
**Total**	24.24	79					

Abbreviations: df, Degree of freedom; F, one way analysis of covariance; MS, mean of squares; Sig, significance; R, regression coefficient; R2, coefficient of determination; SS, Sum of Squares

As shown in *Table 3*, (F = 9.15, R = 0.32, P < 0.01) family flexibility accounted for 9.3% of the variance in food preoccupation. Standardized regression coefficient (Beta) is reported in *Table 4*; the family flexibility (β = 0.324, P < 0.01) was significantly related to food preoccupation, there are multiple associations between family flexibility and food preoccupation, and the 9.3% of the variance of food preoccupation variable is explained by family flexibility.

**Table 4:** Standard Regression Coefficient of Family Flexibility on Food Preoccupation in Crystal Abuser Women

	R	R2	B	βeta	*t*	*P* value
**Prediction**	****	****	****	****	****	******
**Fixed**	-	-	3.83	-	12.66	0.006
**Family flexibility**	- 0.32	0.093	- 0.021	0.324	- 3.02	0.006

Abbreviations: B, unstandardized regression coefficient; R, regression coefficient; R2, coefficient of determination; t, Student’s t – test

As shown in *Table 5*, the family flexibility (F = 0.398, R = 0.07, P < 0.05) was not significantly related to body image, the model has not the ability to predict variance in body image. Because of this inability to predict and explain, that it is not required to report standardized regression coefficient (Beta).

**Table 5:** Summary of the Regression Model of Family Flexibility on Body Image in Crystal Abuser Women

	SS	df	MS	F	P value	R	R2
**Regression**	305.78	1	305.78	0.398	0.53	0.07	0.005
**Residual**	59895.0	78	767.88	-	-	-	-
**Total**	60200.80	79	-	-	-	-	-

Abbreviations: df, Degree of freedom; F, one way analysis of co-variance; MS, mean of squares; R, regression coefficient; R2, coefficient of determination; Sig, significance SS, Sum of Squares

## 5. Discussion

This study was designed to evaluate the association between food preoccupation, body image and family flexibility in crystal abuser women. Family flexibility did not have a significant correlation with body image. The finding of present study is against previous findings which had been performed in this filed such as; Binnenghaven et al. found that Family functioning has a significant relation with body image and body dissatisfaction of daughters (19). In addition, social and cultural variables and family structures many influence body image as well as family functioning does. The result from second question showed that body image did not have a significant association with food preoccupation. According to Park and Beaudet, women below 45 years old who were identified as concerned about their weight were less likely to be preoccupied with food compared to women in the 45 to 64 age group when self-perceived weight, self-esteem and selected socio-demographic characteristics were controlled (13). Because using methamphetamine has devastating effects on their appearance, and since the participants in this research were in the early stages of treatment, it seems, they have compared themselves with the time when they used substance and they have not paid attention to emphasis of researcher to compare with pre-use days and they had a good evaluation of their appearance at that period. Also, effects of obesity and overweight on food preoccupation and body image appear when the weight considered as an unchangeable factor (13, 20). Because some people are reluctant to food intake shortly after taking the substance, then weight is dramatically decreased (1, 2, 5), so that association between food preoccupation and body image was not significant. The results from third question showed that the family flexibility had significant correlation with food preoccupation. Different models and empirical works developed the hypothesis of a disturbance family functioning in eating disorders and behavior of abuse or substance dependence (20). Several studies show that families of patients with eating disorders show a lower adaptability and family cohesion (18, 21-23). Vidovic et al. found that 76 patients with eating disorders had less cohesive and flexible families and also their communication with their mothers was impaired (16). Family conflicting situations are predictors of risk of alcohol or drugs dependence in adolescence (18). Family inability to modify in different situations, leads to family rigidity. So, family responses to stress in stereotypical and inappropriate ways and uses old patterns to deal with new situations. Therefore, all family members participate in futile interactive model and each member plays a role in the preservation and continuation of this disorder, so that, the disease symptoms has an important role in maintaining balance in family (24). It can be argued that flexibility in transitions between stages in the family life cycle, especially during adolescence, have a significant role in shaping eating disorder behaviors. The results from fourth question showed that family flexibility accounted for 9.3% of the variance in food preoccupation, but was not significantly related to body image. Studies have shown associations between poor family functioning and higher depressive symptoms, less academic success, more high-risk behaviors, and more disordered eating behaviors in adolescents. Few studies evaluating the association between family functioning and youth weight and behaviors of potential relevance for weight status have mainly shown poor family communication (25) and lower family functioning (26, 27). Findings of previous studies (25-27) indicate associations between higher family functioning and positive health behaviors (e.g., fruit and vegetable intake, family meals, breakfast consumption, physical activity) and fewer unhealthful behaviors (e.g. sedentary behaviors) in adolescents. The current study indicates that family functioning may be a small, but relevant, correlate of adolescent weight and weight-related health behaviors. Girls with higher family functioning had a lower risk for substance use and disordered eating among other adolescents in previous research (28, 29). According to Berge et al. control behavior, communication, affective involvement, affective expression, and coherence about values and norms seem to be the most relevant dimensions of family functioning. Control within the family describes the process by which individual family members influence or balance each other (20). Effective communication, positive affective expression, and social involvement indicate the extent and the quality of interest in which family members have with each other and the way they express it. The degree of coherence within the family about socially transmitted values and norms also seems to be associated with body image–related problems, impaired functioning in the domains described above can be either a trigger or a result of body image–related problems (20, 23, 26). The process of developing an eating disorder would potentially have a negative influence on family communication and would challenge values and norms. Controlling behavior by family members might be initiated to prevent patients from further damaging their health, and involvement will possibly become more negative. Reciprocal effects are equally imaginable. A controlling family environment with negative communication styles and negative expression and involvement, together with discrepancies on values and norms within the family, might be risk factors for the development of body image-related problems (20). Family functioning seems indeed to play an essential role in body image-related problems (17, 20, 26). However, confrontation of early models of family in the investigation empirical family led to contradictory results, in fact, in some studies there are no significant differences between families of patients with eating disorders and control families, (18, 30, 31). So it can be argued that these results are in concordance with our results which the level of family flexibility is a predictor of food preoccupation, while it is not a predictor of body image. This means that previous research has concluded conflicting findings regarding these associations. Based on these results and previous researches, it is suggested that women’s awareness of the harmful effects of crystal would prevent them to use substance as impotence drug, hence the media has critical role in community awareness. Also, one of the factors which influence substance use is family, therefore family awareness and parent training to increase flexibility is an important role to prevent drug use. One of the limitations of this study is lack of required testing for crystal abuser.

## References

[1] Shrem MT, Halkitis PN (2008). Methamphetamine abuse in the United States: contextual, psychological and sociological considerations.. J Health Psychol..

[2] Homer BD, Solomon TM, Moeller RW, Mascia A, DeRaleau L, Halkitis PN (2008). Methamphetamine abuse and impairment of social functioning: a review of the underlying neurophysiological causes and behavioral implications.. Psychol bulletin..

[3] Harrop EN, Marlatt GA (2010). The comorbidity of substance use disorders and eating disorders in women: prevalence, etiology, and treatment.. Addict Behav..

[4] Root TL, Pisetsky EM, Thornton L, Lichtenstein P, Pedersen NL, Bulik CM (2010). Patterns of co-morbidity of eating disorders and substance use in Swedish females.. Psychol Med..

[5] Dluzen DE, Liu B (2008). Gender differences in methamphetamine use and responses: a review.. Gender Med..

[6] Embry D, Hankins M, Biglan A, Boles S (2009). Behavioral and social correlates of methamphetamine use in a population-based sample of early and later adolescents.. Addict Behav..

[7] Dadsetan P (2207). Psychological development from childhood to adulthood..

[8] Pasha G, Naderi F, Akbari S (2008). Comparison Of Body Image, Body Build Index, General Health And Self Concept Between Beauty Surgery Those Who Have Done Beauty Surgery And Ordinary People In Behbahan.. New Find In Psychol..

[9] Zarshenas S, Karbalaaei Noori A, Hoseini SAR M, Seyednour R, Moshtagh N (2010). The Effects of Aerobic Exercise on Body Image Attitudes in Women.. J Rehabil..

[10] Sarwer DB, Grossbart TA, Didie ER Beauty and society.

[11] Eichen DM, Conner BT, Daly BP, Fauber RL (2012). Weight perception, substance use, and disordered eating behaviors: comparing normal weight and overweight high-school students.. Journal of youth and adolescence..

[12] Tapper K, Pothos EM (2010). Development and validation of a Food Preoccupation Questionnaire.. Eat Behav..

[13] Park J, Beaudet MP (2007). Eating attitudes and their correlates among Canadian women concerned about their weight.. European eating disorders review : J Eat Disord Assoc..

[14] Chandler NG (2010). Parenting style as a moderator for parenting-child food preoccupation.. J Dev Psychol..

[15] Olson DH (1991). Three-dimensional (3-D) Circumplex Model and revised scoring of FACES III.. Family Process..

[16] Vidović V, Jureša V, Begovac I, Mahnik M, Tocilj G (2005). Perceived family cohesion, adaptability and communication in eating disorders. European eating disorders review.. J Eat Disord Assoc..

[17] Minuchin S, Nichols MP, Lee WY (2007). Assessing families and couples: From symptom to system. 1, editor:.

[18] Doba K, Nandino J.L (2010). Is there a family typology of addictive behaviors? Critical review of the literature in the families of adolescents with an eating disorder or with a substance-dependence?. Psychol Française..

[19] Benninghoven D, Tetsch N, Kunzendorf S, Jantschek G (2007). Body image in patients with eating disorders and their mothers, and the role of family functioning.. Compre Psychiatry..

[20] Berge JM, Wall M, Larson N, Loth KA, Neumark-Sztainer D (2012). Family Functioning: Associations With Weight Status, Eating Behaviors, and Physical Activity in Adolescents.. J Adoles Health..

[21] Latzer Y, Lavee Y, Gal S (2009). Marital and Parent—Child Relationships in Families With Daughters Who Have Eating Disorders.. J Family Issues..

[22] Yanez AM, Peix MA, Atserias N, Arnau A, Brug J (2007). Association of eating attitudes between teenage girls and their parents.. Int J Soci Psychiatry..

[23] Kluck AS (2010). Family influence on disordered eating: the role of body image dissatisfaction.. Body image..

[24] Goldenberg I, Goldenberg H (2010). Family therapy..

[25] Chen JL, Kennedy C (2005). Factors associated with obesity in Chinese- American children.. Ped Nurs..

[26] Wen LM, Simpson JM, Baur LA, Rissel C, Flood VM (2011). Family functioning and obesity risk behaviors: implications for early obesity intervention.. Obesity (Silver Spring)..

[27] Zeller MH, Reiter-Purtill J, Modi AC, Gutzwiller J, Vannatta K, Davies WH (2007). Controlled study of critical parent and family factors in the obesigenic environment.. Obesity (Silver Spring)..

[28] Eisenberg ME, Neumark-Sztainer D, Fulkerson JA, Story M (2008). Family meals and substance use: is there a long-term protective association?. J Adol Health..

[29] Burgess-Champoux TL, Larson N, Neumark-Sztainer D, Hannan PJ, Story M (2009). Are family meal patterns associated with overall diet quality during the transition from early to middle adolescence?. J Nutr Edu Behav..

[30] Stradmeijer M, Bosch J, Koops W, Seidell J (2000). Family functioning and psychosocial adjustment in overweight youngsters.. Int J Eat Disord..

[31] De Bourdeaudhuij I, Van Oost P (2000). Personal and family determinants of dietary behaviour in adolescents and their parents.. J Psychol health..

